# Ingested Foreign Bodies Removed by Flexible Endoscopy in Pediatric Patients: A 10-year Retrospective Study

**Published:** 2014-07

**Authors:** Seyed Ali Jafari, Maryam Khalesi, Simin Partovi, MohammadAli Kiani, Hamid Ahanchian, HamidReza Kianifar

**Affiliations:** 1 *Department of Pediatric Gastroenterology, Ghaem Hospital, Mashhad University of Medical Sciences, Mashhad, Iran.*; 2 *Department of Pediatrics, Ghaem Hospital, Mashhad University of Medical Sciences, Mashhad, Iran.*; 3 *Department of Pediatric Immunology, Ghaem Hospital, Mashhad University of Medical Sciences, Mashhad, Iran.*

**Keywords:** Endoscopy, Ingestion, Pediatric

## Abstract

**Introduction:**

Determination of type and location of trapped objects and endoscopic observations among children with foreign-body ingestion.

**Materials and Methods:**

We evaluated 105 endoscopic records of patients presenting with foreign-body ingestion from 2001–2011.

**Results:**

Button batteries were the most common objects removed (41%). The lower segment of the esophagus was the most common trapping site. There was significant correlation between type of foreign body and its location of trapping. Abnormal endoscopic observations were reported in 33% patients. There was significant correlation between the type of foreign body and endoscopic observations. There was also a significant correlation between the location of the foreign body and endoscopic observation.

**Conclusion:**

The pattern of foreign-body ingestion is somewhat different in our center compared with other studies. Awareness among parents about the prevention of this accident is an important step in decreasing the incidence of foreign-body ingestion.

## Introduction

Ingestion of a foreign body is a serious health problem in pediatric patients that causes significant morbidity and mortality. Children have a natural tendency to place objects in their mouth. Approximately 10% of children appear to be recidivists (1,2) . 

Serious complications from foreign-body ingestion have been reported by many authors, including sudden death, esophageal perforation, fistula, and abscess formation (3,4).

The most common ingested foreign bodies have included coins, toy parts, jewels, batteries, fish, chicken bones, large amounts of food, and sharp materials such as needles and pins (1). 

To the best of our knowledge, no specific investigation has been conducted to explore endoscopic removal of foreign bodies in the pediatric population in Iran. Currently available data are derived from worldwide epidemiological investigations about foreign-body ingestion.

This study investigated foreign-body ingestion treated with an endoscopic procedure in Ghaem Hospital of Mashhad University of Medical Sciences over a 10-year period in pediatric patients (less than 15 years).

## Materials and Methods

We identified the endoscopic records of patients with foreign-body ingestion between 2001 and 2011 from the Endoscopic Department of Pediatric Gastroenterology, Ghaem hospital, Mashhad, Iran. Patients aged less than 15 years with a diagnosis of foreign-body ingestion who had undergone endoscopic removal were included. Patients were excluded if they spontaneously excreted the foreign body, as confirmed by observation. For foreign bodies trapped in the esophagus, removal was performed immediately if the patient was symptomatic or if the foreign body was dangerous, and after 24 hours under other conditions. The waiting time for foreign bodies in the stomach was up to 1 week, except for dangerous or large foreign bodies which were removed as soon as possible.

Demographic data, clinical presentation, radiological findings, type of foreign body, location in the gastrointestinal tract, and endoscopic findings (normal or abnormal and the location of abnormality) were recorded.

We analyzed the results using the chi-square test (2%). A *p-*value of less than 0.05 was considered significant.

## Results

One hundred and five patients with a diagnosis of foreign-body ingestion underwent endoscopic removal. The mean (range) age of the patients was 4 years (8 months to 14 years). Males were affected more than females (male/female ratio: 2/1). Button batteries (41%), needles (25%), coins (16%), and food-related items (15%) were the most commonly removed objects. Other objects included toy particles, metallic plaques, glass, and wood pieces. Demographic, clinical, and radiological findings are summarized in Table 1. 

Objects were predominantly located in the lower segment of the esophagus. There was significant correlation between the type of foreign body and its location (Table 1). 

**Table1:** Demographic, clinical and radiological findings of patients

			**No (%)**
Sex	Female		36 (34)
Age (years)	Mean (range)		4(8 months-14 y)
Clinical symptoms	No symptom Dysphagia Vomiting Drooling Abdominal pain Chest pain GI bleeding coughing		31 (30) 44 (42) 37 (35) 34 (33) 5 (5) 12 (12) 3 (3) 27 (26)
Radiological Findings	Non opaque Opaque		41 (39) 64(61)

Thus, the lower esophageal segment was the most common trapping site for button batteries (57.9%) and food-related items (75%), while the stomach and duodenum were the most common trapping sites for needles (66%). Coins were trapped equally in the lower esophageal segment (33%) and the stomach (33%). We observed abnormal endoscopic observations in 33% of patients, including mucosal erythema, erosion, ulcer, and necrosis. There was a significant correlation between the type of foreign body and endoscopic observations (Table 2). The most common abnormal endoscopic observations were associated with food-related items (50%) and button batteries (45%). 

**Table 2:** Type and location of foreign bodies in the gastrointestinal tract and endoscopic observations

**Type of foreign body**	**Location of foreign body, Number**					**Endoscopic** ** observations, number**	
	Upper esophageal	Mid-esophageal	Lower esophageal	Stomach	Duodenum	Normal	Abnormal
Button battery	3	11	24	4	0	22	20
Needle	3	3	2	9	9	21	5
Coin	3	3	6	6	0	16	2
Fragment of food	2	2	12	0	0	8	8
Others	0	0	1	2	0	36	0
Total	11	19	45	21	9	70	
							

There was also a significant correlation between the location of the foreign body and the endoscopic observations. Foreign bodies trapped in the lower or middle segment of the esophagus were most likely to show abnormalities on endoscopic observation (48% and 47%, respectively). The upper esophageal segment (18%), duodenum (12%), and stomach (5%) were the next were the next most likly to show endoscopic abnormality (Table 3).

**Table 3:** Endoscopic observations according to location of foreign body in the gastrointestinal tract

**Location of foreign body**	**Endoscopic observations, Number**	
	Normal,no(%)	Abnormal,no(%)
Upper esophageal	9(82)	2(18)
Mid-esophageal	10(53)	9(47)
Lower esophageal	23(52)	22(48)
Stomach	20(95)	1(5)
Duodenum	8(88)	1(12)
Total	70	35

## Discussion

Several authors have highlighted differences between Western and Asian pediatric foreign-body injuries, suggesting that environmental and ethnic food habits may influence the age distribution and type of esophageal foreign bodies (5-8). 

In our center, button batteries were the most common foreign bodies removed (41%), compared with coins in other studies (9-11). Because of financial policies in our country, the use of coins as currency is not as common as previously. This may be the reason for this difference. In a study by Theologos et al., battery ingestion accounted for 4% of all inedible foreign-body ingestions compared with 12% for coins (10). Gregori et.al reported that batteries accounted for 2.8% of all injections compared with 54% for coins (11). Consistent with our results, button battery ingestions have been associated with a high incidence of abnormal endoscopic findings in several studies. 

Button battery-related injuries resulted from direct pressure necrosis, alkali leakage, and local electrical currents (12-14).

Button batteries are commonly used in toys, digital watches, musical greeting cards, and hand-held calculators. Use of toys and other products with a safe and tightly secured battery container may be the most important method for prevention. Children cannot remove the battery, and the battery will not be released if the product is dropped. 

 Food-related items were the least common ingested foreign bodies (15%). There are various reports considering food ingestion in different studies. Similar to our findings, Georgio et al. reported that 15% of foreign-body ingestions were related to food (11). Due to eating habits in a number of Asian communities, fish-bone ingestion is particularly common. In the study by Kennet et.al, fish bone was the most common ingested foreign body (68.8%) (4). Food impaction usually occurs in children with an esophageal pathology (e.g., stricture, achalasia, neuromuscular disease, or eosinophilic esophagitis) (1,2). It is important to mention that despite the high incidence of abnormalities at endoscopy, the most common endoscopic abnormalities were simple mucosal erythema related to fragmented food trapping; the more serious endoscopic abnormalities of ulcer or necrosis were associated with the less common button battery ingestion.

In a study by Little et al, the upper segment of the esophagus was the most common trapping site (73%) (13). The location of the foreign body in the gastrointestinal tract is dependent on the size of the foreign body. We also recorded the location of the foreign body during the endoscopic procedure. Endoscopic removal of more dangerous foreign bodies (e.g., button batteries) from the esophagus rather than less dangerous ones (e.g., coins) from stomach allows the opportunity for spontaneous safer excretion.

This study was performed retrospectively on the basis of endoscopic records. A prospective long-term study with regard to risk factors for foreign-body ingestion, demographic data, presented signs and symptoms, radiographic findings, and follow-up for complications would provide further valuable information.

## Conclusion

It is important to increase awareness among parents about the ingestion risks posed by some objects, especially button batteries, in order to diminish the incidence of foreign-body ingestion and its related sequelae.
