# Correction: Ingested Foreign Bodies Removed by Flexible Endoscopy in Pediatric Patients: A 10-year Retrospective Study

**Published:** 2014-10

Seyed Ali Jafari, Maryam Khalesi, Simin Partovi, MohammadAli Kiani, Hamid Ahanchian, HamidReza Kianifar. Ingested Foreign Bodies Removed by Flexible Endoscopy in Pediatric Patients: A 10-year Retrospective Study. Iran J Otorhinolaryngol. 2014;26(76):175–179.

The correct Title is provided. On page 175, title, lexible should be Flexible.

